# Mass spectrometry quantitation of proteins from small pools of developing auditory and vestibular cells

**DOI:** 10.1038/sdata.2018.128

**Published:** 2018-07-17

**Authors:** Jocelyn F. Krey, Deborah I. Scheffer, Dongseok Choi, Ashok Reddy, Larry L. David, David P. Corey, Peter G. Barr-Gillespie

**Affiliations:** 1 Oregon Hearing Research Center & Vollum Institute, Oregon Health & Science University, Portland, OR 97239, USA; 2 Department of Neurobiology, Harvard Medical School, Boston, MA 02115, USA; 3 OHSU-PSU School of Public Health, Oregon Health & Science University, Portland, OR 97239, USA; 4 Graduate School of Dentistry, Kyung Hee University, Seoul, Korea; 5 Department of Biochemistry and Molecular Biology, Oregon Health & Science University, Portland, OR 97239, USA; 6 Proteomics Shared Resource, Oregon Health & Science University, Portland, OR 97239, USA

**Keywords:** Cell type diversity, Inner ear, Mass spectrometry, Proteomic analysis

## Abstract

Hair cells of the inner ear undergo postnatal development that leads to formation of their sensory organelles, synaptic machinery, and in the case of cochlear outer hair cells, their electromotile mechanism. To examine how the proteome changes over development from postnatal days 0 through 7, we isolated pools of 5000 Pou4f3-Gfp positive or negative cells from the cochlea or utricles; these cell pools were analysed by data-dependent and data-independent acquisition (DDA and DIA) mass spectrometry. DDA data were used to generate spectral libraries, which enabled identification and accurate quantitation of specific proteins using the DIA datasets. DIA measurements were extremely sensitive; we were able to detect proteins present at less than one part in 100,000 from only 312 hair cells. The DDA and DIA datasets will be valuable for accurately quantifying proteins in hair cells and non-hair cells over this developmental window.

## Background & Summary

Hair cells, the sensory cells of the inner ear, are responsible for detection, amplification, and transmission of auditory and vestibular stimuli^1^. In this context, the “hairs” are microscopic cilia, composed mostly of actin rather than keratin, and are key structures in the ear that sense sound and enable balance. In rodents, most of the key developmental steps in hair cell development occur in the first two postnatal weeks^2,3^. While key participants in critical hair cell functions have been identified by various means, the full developmental programs that produce a functional hair cell are poorly understood.

Dissecting the steps in hair cell development has been impeded by the small number of cells and complexity of the mammalian inner ear’s structure. In a mouse cochlea, which mediates auditory transduction, ~3000 hair cells reside in the organ of Corti, a small organ of about a dozen cell types that sits on the vibrating basilar membrane^4,5^. By contrast, the sensory epithelium of a single utricle, one of the several organs responsible for vestibular transduction, is a more simply constructed checkerboard of ~4000 hair cells and a single class of supporting cell^6^. Regardless, the intermixing of cell types in each organ complicates molecular analysis of hair cell development.

The first step in a molecular description of hair cell development is the determination of the time course of expression of key molecules. As precursors to proteins are relatively easy to measure, transcripts’ developmental profiles are often measured first. Expression of key transcripts in mouse cochlear and utricular hair cells has been evaluated by fluorescence-activated cell sorting (FACS) of cells, followed by RNA-seq^7,8^. The *Pou4f3-Gfp* mouse line, which expresses green fluorescent protein (GFP) under control of the hair-cell-specific *Pou4f3* promoter, allowed selected sorting of hair cells away from all other cells in the cochlea and utricle. Analysis of transcripts enriched in hair cells at the developmental time points embryonic day 18 (E18) through postnatal day 7 (P7) allowed identification of several molecules essential for cochlear and utricular development^7,9^.

While measurement of transcript levels in pooled, sorted cells is extremely sensitive, proteins are the effectors of a cell. Indeed, the correlation between transcript level and protein abundance is weak at best^10,11^, and direct measurement of protein levels is desired. Experiments using the hair-cell dye AM1-43 to label chicken utricle and saccule hair cells for FACS sorting led to identification and quantitation of ~600 proteins but required ~170,000 hair cells from ~120 inner-ear organs per experiment^12^. The *Pou4f3-GFP* mouse line has also been used for measurement of protein abundance in experiments similar to the transcript analysis described above; mass spectrometry analysis of large pools of hair cells sorted by FACS allowed the identification of >6000 proteins, including >900 specifically expressed in hair cells^13^. This exceptional depth of analysis required ~200 000 hair cells per sample^13^. However, since >100 animals were required and vestibular and cochlear hair cells combined, the experimental questions that could be asked were limited. For example, examining the developmental progression of proteins enriched in hair cells separately in cochlear and vestibular tissues would have required an extremely large number of animals if this large number of cells per sample was maintained.

We instead devised methods to examine proteins in much smaller numbers of pooled, sorted cells at P0, P4, and P7. To complement previous transcript and protein analyses on sorted cells, we isolated hair cells from *Pou4f3-Gfp* cochleas and utricles and carried out mass-spectrometry analysis of the proteins present. Because its paired quadrupole and Orbitrap modules allowed efficient isolation of precursor peptides and highly accurate detection of fragmentation products, we used a Q Exactive HF mass spectrometer to measure protein abundance in sorted cells using both data-dependent and data-independent acquisition (DDA and DIA) strategies^14^. DDA is valuable for measuring the breadth of protein expression in isolated cells and to identify peptides suitable for DIA analysis, while DIA provides accurate relative abundance measurements for proteins present in isolated cells. The mass spectrometer’s sensitivity allowed us to measure three time points in duplicate (DDA) and triplicate (DIA), separately for cochlea and utricle GFP-positive and -negative cells, using only 5000 sorted cells per replicate. The coupled DDA and DIA datasets will be valuable resources for measuring the dynamics of protein expression, or expression in auditory vs. vestibular cells, for any protein that can be identified in DDA datasets.

## Methods

### Isolation of hair cells and inner-ear tissue from *Pou4f3-Gfp* mice

**Figure 1:**
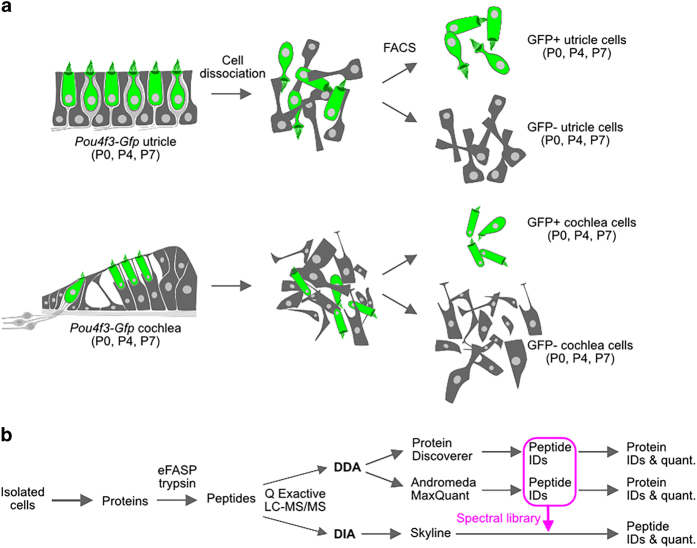
Samples and workflow. **a**, Utricles and cochleas with hair cells marked with GFP were subject to cell dissociation and FACS at three different developmental ages. GFP-positive or GFP-negative cells were separately collected and used for further analysis. **b**, Pools of 5000 isolated cells collected under the appropriate conditions were denatured, and their proteins were digested to peptides using the protease trypsin and the eFASP preparation method. Identical aliquots were run on a Q Exactive HF mass spectrometry either using data-dependent (DDA) or data-independent (DIA) acquisition.

Methods used for isolating single cells from the inner ear have been described in detail elsewhere^7^ and are illustrated in Fig. 1. To selectively isolate hair cells, we used animals of either sex from the Tg(*Pou4f3-8.5-eGFP*) transgenic mouse line, which expresses enhanced GFP under control of the *Pou4f3* promoter^15^; the high specificity of the *Pou4f3* promoter ensures that the only labelled cells are hair cells. This mouse line was obtained from the laboratory of Dr. Allen Ryan (University of California San Diego). Utricles and cochleae were dissected in less than 1 hr using ice-cold PBS, then were transferred to ice-cold DMEM (Life Technologies) with 5% FBS. To dissociate the cells, organs were treated at 37 °C in 1 mg/ml Dispase (Gibco) and 1 mg/ml collagenase I (Worthington) in 100 μl for batches of 10-12 utricles or 200 μl for batches of 10-12 cochleae. Digestion was allowed to proceed for 30 min at P0, or for 45 min at P4 and P7. Dissociation was carried out by triturating with a pipette, and the extent of dissociation was observed with an inverted microscope. Dissociation was completed in dissociation buffer (Gibco 13151–014, with 5% FBS) and the samples transferred to ice. To eliminate clumps before sorting, dissociated cell suspensions were filtered through a cell strainer with a 40 μm mesh. Cells were sorted on a BD FACS Aria II cell sorter using a 100 μm nozzle and low pressure. Hair cells were collected using the brightest GFP fluorescence signal and other cells were collected using the lowest fluorescence signal. Cells were counted and collected in aliquots of 1000-10,000 cells directly into PBS and were frozen at −80 °C before mass spectrometry sample preparation and analysis. All cell isolation was carried out at Harvard and cell samples were shipped on dry ice to OHSU.

### Sample preparation for mass spectrometry

Cell aliquots were pooled and diluted if necessary to create samples of ~5000 GFP-positive or negative cells per condition. Cochlear hair cells are 0.5–1.0 pl in volume^16,17^; if their total protein concentration was ~200 mg/ml (refs 18,19), each cell would contain 0.1–0.2 ng of protein. Prior to sample preparation, each sample’s 5000 hair cells together are thus predicted to contain 0.5-1.0 μg of total protein. The GFP-negative cells are likely somewhat larger in size^16,17^ and thus have larger amounts of total protein.

In-solution tryptic digests of the samples were prepared using an enhanced filter-aided sample preparation (eFASP) method^20^. Cells were solubilized in lysate buffer, which contained 4% SDS, 0.2% deoxycholic acid (DCA), 50 mM TCEP, and 100 mM ammonium bicarbonate at pH 8.0. Lysis was carried out at 90 °C for 10 minutes. The proteins were then exchanged into a urea buffer, which contained 8 M urea, 0.2% DCA, and 100 mM ammonium bicarbonate (AB), using a series of spins in a 0.5 ml 30 kDa cut-off filter unit (Amicon Ultra) that had been passivated in a 5% Tween-20 solution the day before. Alkylation solution (8 M urea, 50 mM iodoacetamide, 100 mM AB) was then added to the filter unit and tubes were mixed for 1 h at 37 °C. Proteins were then exchanged into digestion buffer (0.2% DCA and 50 mM AB) and digested in the filter unit in 100 μl digestion buffer with 200 ng sequencing-grade modified trypsin (Promega) at 37 °C for 12–16 h. Peptides were isolated by centrifugation and were extracted with ethyl acetate to remove remaining deoxycholic acid^20^.

### Mass spectrometry: data-dependent acquisition

Protein digests were separated using liquid chromatography with a NanoAcquity UPLC system (Waters); analytes were ionized using electrospray with a Nano Flex Ion Spray Source (Thermo Fisher) fitted with a 20 μm stainless steel nano-bore emitter spray tip and 1.8 kV source voltage, and were delivered to a Q Exactive HF (Thermo Fisher). Xcalibur version 4.0 was used to control the system. Samples were first bound to a trap cartridge (Symmetry C18 trap cartridge; Waters) at 15 μl/min for 10 min; the system then switched to a 75 μm x 250 mm NanoAcquity BEH 130 C18 column with 1.7 μm particles (Waters) using mobile phases water and acetonitrile containing 0.1% formic acid. A 7.5–30% acetonitrile gradient was delivered over 60 min at a flow rate of 300 nl/min. Survey mass spectra were acquired in m/z 395−1005 at 120,000 resolution (at 200 m/z); data-dependent acquisition selected the top 10 most abundant precursor ions for tandem mass spectrometry using an isolation width of 1.2 m/z. HCD fragmentation used normalized collision energy of 26 and a resolution of 30 000. Dynamic exclusion was set to auto, charge state for MS/MS +2 to +7, maximum ion time 100 ms, minimum AGC target of 3×10^6^ in MS1 mode and 5 x 10^3^ in MS2 mode. DDA data were searched with Andromeda^21^ and analysed using MaxQuant^22^ version 1.5.3.12 with peptide and protein false-discovery rates of 1%; these data were also used for generating the DIA spectral library. DDA data were also searched with Protein Discoverer (Thermo Fisher) using Sequest HT, a modified form of the original SEQUEST search algorithm^23^; the Discoverer output also contributed to the spectral library. We used the Ensembl *Mus musculus* GRCm38.71 database (released April 2013) with the addition of XIRP2 splice products and 179 common contaminants. The database included 50 878 forward and 51 056 reversed entries. For database searching, we specified trypsin/P cleavage, no more than two missed cleavages, and allowed methionine oxidation and N-terminal acetylation.

### Mass spectrometry: data-independent acquisition

Protein digests were separated using liquid chromatography and ionized under the same conditions used for DDA. The DIA mass spectrometry method was constructed using 20 m/z quadrupole isolation windows. First, a full scan at 30,000 FWHM resolving power (at 200 m/z) was performed, followed by sequential HCD-MS/MS scans at normalized collision energy of 26 and 15,000 FWHM resolution. The range between 395 and 1005 m/z was surveyed by these experiments with maximum injection times of 55 ms for MS and the “auto” setting for MS/MS. AGC values were set to 3×10^6^ for MS and 1×10^6^ for MS/MS. The MS/MS scan range was set to 200–2000 m/z. DIA data were analysed with Skyline^24,25^ version 3.7.

For global normalization, we computed the median of protein quantities in each sample, m_i_, for i=1 to n, where n is the total number of samples. We then computed the overall median of the medians (global median). Finally, for x_ij_, the quantity of the j-th protein in sample i, we computed normalized data using y_ij_=x_ij_ – m_i_+ global median for j=1 to k, where k is the number of peptides in sample i. For fractional normalization, for each sample, we summed the intensities for all peptides; each peptide’s intensity was then divided by the sum of intensities.

## Data Records

All DDA data and analysis files (described below), as well as the DIA data, have been deposited to ProteomeXchange (Data Citation 1). This dataset includes 97 binary instrument files (.RAW), representing LC-MS/MS data from the twelve principal experimental conditions (P0, P4, and P7; cochlea and utricle cells; GFP-positive and GFP-negative cells), as well as runs analysing whole utricle, whole cochlea, and isolated utricle hair bundles (Data Citation 1). The dataset also includes "SEARCH.zip", which contains all of the MaxQuant files from the "txt" folder, as well as the "experimentalDesignTemplate.txt" file that specifies how the files are to be searched by MaxQuant (Data Citation 1). "SEARCH.zip" also includes results from Protein Discoverer searches of the DDA data. "OTHER.zip" (Data Citation 1) contains the FASTA file used for the MaxQuant search (Mus_musculus.GRCm38.71.pep.all.fixed_both.added. Xirp2.Gm1322), as well as files listing Minimal Information About a Proteomics Experiment^26^ for the DDA and DIA runs, the MaxQuant “conf” folder, and a folder of Skyline spectral libraries and actin capping protein results^27^.

All DIA analysis files (described below) have been published to Panorama Public, an online repository for Skyline documents^28^. The files can be viewed and downloaded using the following link: https://panoramaweb.org/labkey/pou4f3FACsDIA.url. The dataset includes three full Skyline files that contain the selected peptides and proteins chosen for analysis, the chromatograms from each of the twelve experimental conditions, and the spectral library generated from the search results for the DDA data (see above).

## Technical Validation

### DDA development samples

As described in Methods and Usage Notes, we used MaxQuant to identify and quantify proteins from the DDA data (Fig. 1b); for each protein or protein group, MaxQuant reports an intensity-based absolute quantification (iBAQ) value, a measure of protein abundance^29^. In our initial analysis, for each protein, we normalized the data by linearly calculating abundance relative to the total. We determined the relative iBAQ (riBAQ), which is the iBAQ for a protein or protein group (calculated by MaxQuant) divided by all non-contaminant, non-reversed iBAQ values for a replicate. riBAQ is equivalent to normalized molar intensity^30^ (*i*), and is an example of a transformation we call “fractional normalization.” These data are available at *Figshare* (File “Krey, Choi, and Barr-Gillespie Table 1 DDA analysis (all proteins)”, Data Citation 2)^31^.

**Figure 2:**
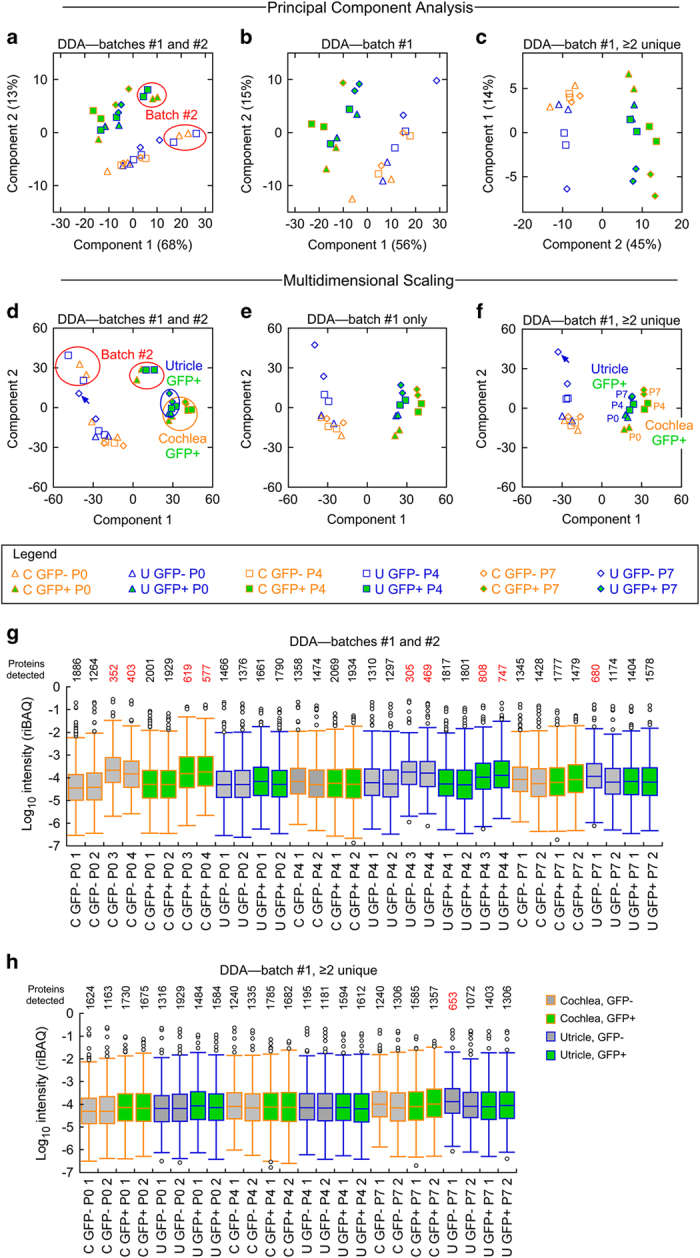
Analysis of DDA samples. **a–f**, Similarity of DDA samples at the protein level, analysed by principal component analysis (PCA) and multidimensional scaling (MDS). **a**, PCA analysis of all 32 samples analysed by DDA mass spectrometry, each with 5000 cells. Red ovals highlight samples prepared in batch #2. The percentage of the variance contributed by each principal component is indicated in the axis. **b**, PCA analysis of the 24 vetted DDA samples. Note that the samples cluster together more tightly than in A. **c**, PCA analysis of the 24 vetted DDA samples, reanalysed with the requirement of two or more unique peptides supporting each identification. **d**, MDS analysis of all 32 samples analysed by DDA mass spectrometry. **e**, MDS analysis of the 24 vetted DDA samples. **f**, MDS analysis of the 24 vetted DDA samples, reanalysed with the requirement of two or more unique peptides supporting each identification. Note the close correspondence of each pair of replicates, with the exception of one of the P7 utricle GFP-negative replicates (blue arrow in panels D-F). The largest variance is associated with GFP state and the second variance component separates samples by developmental time. Legend in F applies to all panels. **g**,**h**, Distribution of riBAQ values for proteins in each DDA sample. Boxes contain the upper (UQ) and lower quartiles (LQ), with the median value indicated by a horizontal line and the interquartile distance (IQD) being UQ-LQ. The remainder of the data are within the lines, with the exception of outliers (defined as >UQ+1.5•IQD or <LQ-1.5•IQD), plotted as individual points. The numbers on the top indicate the total number of proteins or protein groups detected in each sample; the value is coloured red if <1000.

Each experimental condition had two or four biological replicates, which were prepared in two batches. After log transformation of the data, principal-component analysis (PCA)^32^ showed substantial variability in the distribution of protein abundance between, but also within, sample conditions (Fig. 2a). Likewise, analysis using classical multidimensional scaling (MDS), based on principal coordinates analysis^33^, showed both clear separation of GFP-positive samples from GFP-negative samples, but also dispersion of replicates (Fig. 2d). Both methods are widely used to project high dimensional data onto 2- or 3-dimensional space. PCA transforms the (correlated) original variables into uncorrelated variables (principal components); typically, the first few components contain the majority of information contained in the original variables. By contrast, the classical MDS is based on the principal coordinates that represent the degree of similarity of individual samples as well as possible in a low dimension.

We noted that nine samples had fewer than expected numbers of protein identifications than the remaining 23 samples (Fig. 2g). Because eight of the nine samples with reduced protein numbers were processed for mass spectrometry together at the same time and were run together in a group (batch #2; Table 1), we suspected that these samples were less representative than the batch #1 cells. Indeed, both PCA and MDS analyses showed that batch #2 samples segregated from those prepared in batch #1 (Fig. 2a,d). When we removed these eight samples from the analysis, leaving duplicates (replicates #1 and #2) for each experimental condition from sample-preparation batch #1, PCA and MDS analyses indicated that each pair of samples was similar in its pattern of protein expression and that GFP-positive cochlea or utricle cells clustered together (Fig. 2b,e). Thus while PXD006240 contains all 32 of the FACS-sorted cell samples, we only recommend using the 24 samples indicated in Table 1 that derive from batch #1.

The default MaxQuant settings allow for a single peptide to define a protein. When we increased the stringency to require two or more unique peptides for each protein, we detected somewhat fewer proteins overall in each sample (Fig. 2h). Notably, replicates were clustered somewhat closer together (Fig. 2c,f). These data are available at *Figshare* (File “Krey, Choi, and Barr-Gillespie Table 2 DDA analysis (2 unique)”, Data Citation 2).

**Figure 3:**
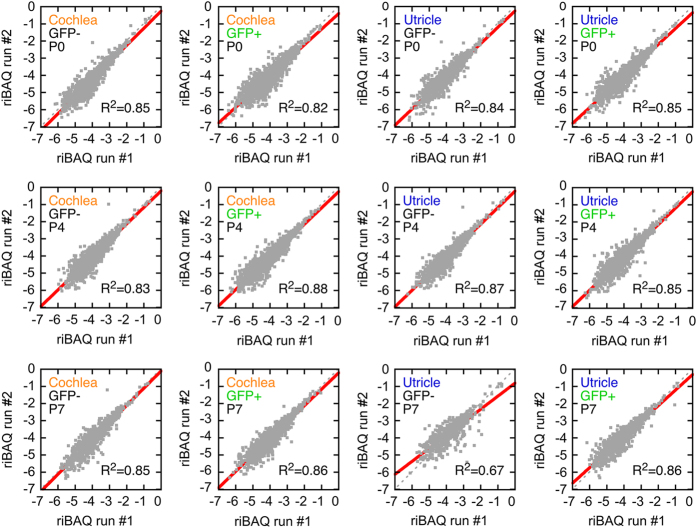
Comparison of protein riBAQ values between biological replicates for each DDA condition. Data were from MaxQuant searches requiring two or more unique proteins to identify a protein. Each point represents one protein detected in both biological replicate #1 and #2 for the indicated conditions. The grey dashed line represents a slope of one, while the red line is a linear fit of the log-log data, with the correlation coefficient indicated.

Using the 24 vetted samples (batch #1) and requiring two unique peptides per identification, pairwise comparisons of individual protein levels for replicates #1 and #2 for each of the sample conditions showed excellent correspondence, with R^2^ values of 0.8–0.9 (Fig. 3; Suppl. Figures 1–3), with the exception of the P7 utricle GFP-negative samples, one of which was already highlighted as having reduced protein identifications.

Although Hickox *et al.*^12^ identified 934 hair-cell-specific proteins in similar FACS-sorting experiments, these were from only 458 genes; multiple Uniprot entries were matched for many proteins. Using protein symbols to compare the entries in Table 1 of Hickox *et al.* with all proteins identified in hair cells from our dataset, we only found 160 of those 458 proteins in our GFP-positive hair-cell samples. Many of the hair-cell-specific proteins identified by Hickox *et al.*, were identified with only a single peptide, however, which may explain the relatively small overlap. Nevertheless, many proteins that are well known to be hair-cell-specific were identified in the set of 160 common proteins (e.g., ESPN, ESPNL, GRXCR1, MSRB3, MYO3A, MYO3B, OCM, POU4F3, SLC26A5, STRC, TOMT, XIRP2).

### DIA development samples

DIA data were analysed with Skyline (Fig. 1b), which can operate in modes that we call “manual” and “automated.” In the manual mode, a spectral library is used to identify precursors and transitions for a limited number of specified proteins. The chromatographic profile is examined manually to determine the correct peaks for each peptide; this step limits the number of peptides that can be quantified. In the automated mode, Skyline uses the mProphet^34^ semi-supervised learning algorithm to identify correct peaks for quantitation. A chromatogram peak scoring model is refined using acquired data, and the model’s coefficients (from peak feature scores) are used to define an mProphet score. The resulting null distribution parameters obtained from the model allow Skyline to estimate q-values for each peptide and hence false-discovery rates (FDR)^35^. The automated mode can be used to identify as many as tens of thousands of peptides per sample. Manual inspection was particularly useful for peptides that were missing in one set of samples, e.g., MYO7A (a highly hair-cell-specific protein) in GFP-negative cells. In the automated mode, Skyline sometimes picked an inappropriate peak when a good one was absent or had poor signal-to-noise, although those inappropriate peaks were usually filtered out because of their poor q-value. However, in the manual mode, we could not carry out standard normalization methods because only a small subset of peptides were quantified. The manual mode is thus most useful for comparisons of a single peptide or protein across multiple conditions, whereas the automated mode allows nearly proteome-wide comparisons.

All samples for DIA were prepared together in triplicate in batch #1 along with the successfully processed DDA samples (Table 2) and were analysed out using the Q Exactive HF in a DIA mode. We initially analysed the DIA dataset using Skyline’s automated method. We chose the 2000 most abundant proteins in the complete DDA dataset to analyse; after the Skyline import, 1872 proteins were present with two or more unique peptides 7-50 amino acids in length. We used a spectral library derived from the MaxQuant DDA data from the twelve developmental, organ, and time point samples; none of the peptide spectra from batch #2 samples were used in this spectral library. We also created an internal retention time (iRT) calculator using 13 conserved common internal retention time (CiRT) standard peptides to generate a retention time predictor for the data^36^. We quantified 14,453 peptides from these 1872 proteins; these data are available at *Figshare* (File “Krey, Choi, and Barr-Gillespie Table 3 DIA analysis (2000 most abundant)” Data Citation 2). We reported an intensity for a given peptide in a sample if it had a q-value (FDR) of 0.01 or less. In addition to extracting fragment-ion intensities, which are often used for DIA or parallel reaction monitoring (PRM) analysis, Skyline also extracts the parent-ion (MS1) intensity for each peptide; both fragment-ion and MS1 intensities are reported in materials available at *Figshare* (File “DIA analysis (2000 most abundant)”, Data Citation 2), along with the library dot product, isotope dot product, and average mass error for each measurement. Information about the mProphet model is available (*Figshare* file “mProphet features (DIA model)”, Data Citation 2).

**Figure 4:**
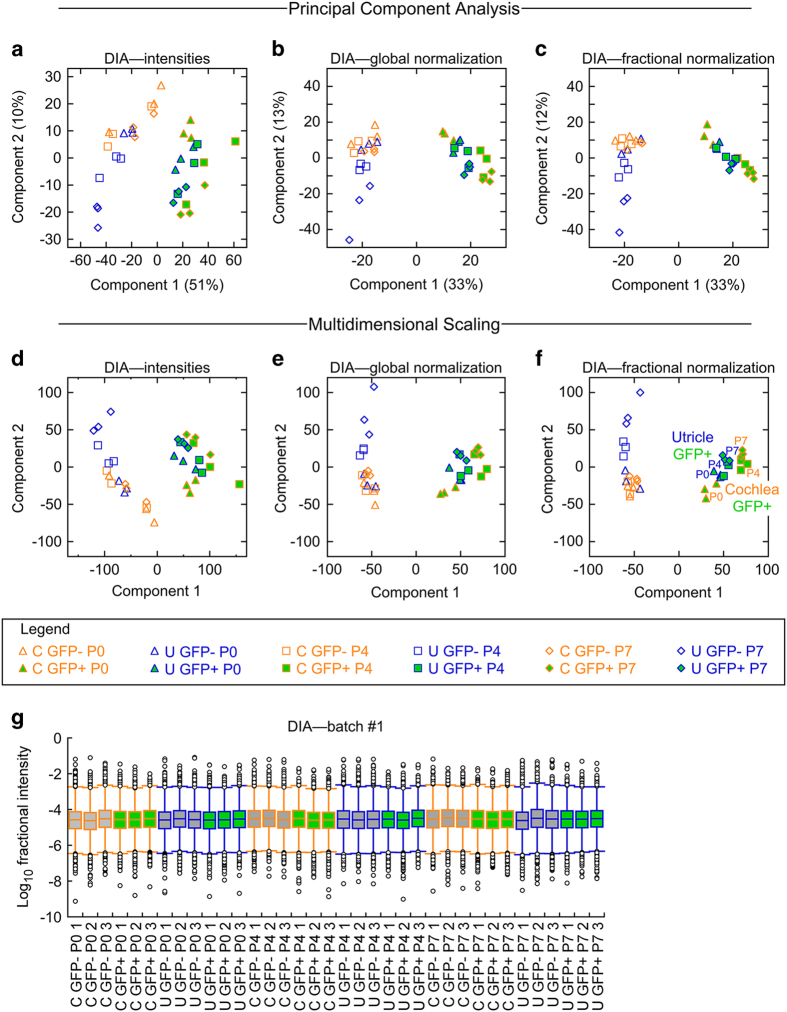
Analysis of DIA samples. **a–f**, Similarity of DIA samples at the peptide level, analysed by PCA and MDS. Each sample contained 5000 cells, and triplicate samples were analysed for each of the 12 conditions. Data for the 1872 most abundant proteins, as defined by DDA analysis, were extracted from the DIA dataset for both PCA (**a–c**) and MDS (**d–f**) analyses. For PCA samples, percentage of the variance contributed by each principal component is indicated in the axis. **a**, **d**: Unnormalized. **b**, **e**: Global normalization. **c**, **f**: Fractional normalization (intensity for a given peptide divided by the sum of all peptide intensities). **g**, Distribution of fractional intensity values for peptides in each DIA sample. Box plot definitions are the same as in Fig. 2e,f.

We again used PCA and MDS to determine whether the replicates for each condition were well matched. When raw MS1 intensities were used for analysis (Fig. 4a,d), sets of triplicate samples clustered reasonably well. However, there was significant sample-to-sample variation in total intensity for all matched peptides (*Figshare* file “DIA analysis (2000 most abundant)”, Data Citation 2), suggesting that data normalization was required. Either global normalization (Fig. 4b,e)^37^ or fractional normalization (Fig. 4c,f) led to more tightly clustered samples in two-dimensional plots for PCA and MDS. Plotting three components for the MDS analysis showed better clustering of samples (*Figshare* files “3D MDS analysis view 1” and “3D MDS analysis view 2”, Data Citation 2). Following fractional normalization, peptide intensity values were distributed similarly for all samples (Fig. 4g).

### Features of DIA data

**Figure 5:**
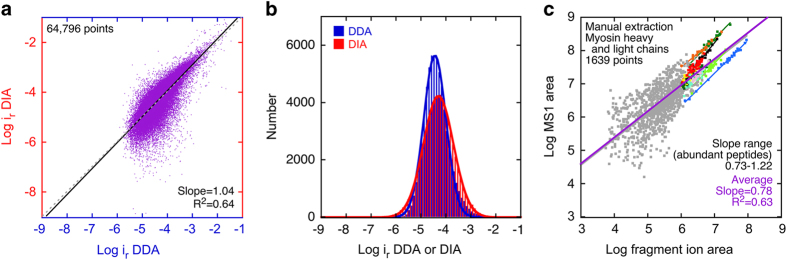
DIA peptide intensity analysis. **a**,**b**, Comparison of DDA and DIA data for individual peptides under each experimental condition. For both DDA and DIA data, intensities for all charge and modification states were summed together for each amino acid sequence. For both DDA and DIA data, MS1 intensities for each condition were normalized by dividing the intensity of each peptide by the sum of all measured peptide intensities. The duplicates (DDA) or triplicates (DIA) were averaged together, then the data was log_10_-transformed. **a**, Relationship between DDA and DIA data. Each of the 64,796 points corresponds to the DDA and DIA results for one amino acid sequence under one experimental condition (cochlea vs. utricle; GFP- vs. GFP+; P0, P4, or P7). The log-transformed data were fit with y=mx+b (red line; m=1.050±0.003, b=0.24±0.01); the unity line is also displayed (grey dashed line). ***b***, Distribution of DDA and DIA values. Binned data were fit with single Gaussian functions. **c**, Comparison of fragment-ion area (intensity) and MS1 area (intensity) for manually-extracted myosins data. For 1639 combinations of peptide and sample, the log_10_ of the fragment-ion area was compared to the log_10_ of the MS1 area. On average, the slope was 0.78, suggesting that MS1 area was lower than fragment-ion area. For ten high-abundance peptides (indicated with coloured points and linear fits), slopes ranged from 0.73 to 1.22, reflecting a substantial peptide-to-peptide variation.

While the above DDA analysis was done at the protein level, MaxQuant reports intensity data for all identified peptides. We therefore determined how similar the DDA and DIA peptide intensities were for each sample condition. Separately for the DDA and DIA data and for each sample condition, we combined all charge and modification states for each peptide, then averaged MS1 intensities across duplicates (DDA) or triplicates (DIA). We then compared the relative intensity values for each peptide under each condition that was detected with both DDA and DIA (Fig. 5). For these 64,797 DDA-DIA pairs, the correlation between the DDA and DIA data was good, especially at the higher intensity levels (Fig. 5a). The distribution of intensity values was somewhat broader for the DIA data than the DDA data (Fig. 5b).

We also used the manually extracted data to determine the relationship between fragment-ion intensity and MS1 intensity (Fig. 5c). There was substantial scatter in this relationship, although individual peptides showed good to excellent linearity (R^2^ for ten abundant peptides ranged from 0.64 to 0.99), with the strongest linearity for peptides that varied substantially between samples and thus were widely distributed in intensity.

### Manual vs. automated data extraction

DIA data can also be extracted manually, which in particular allows for a more conclusive determination of the absence of a peptide in a sample. For comparison of quantitation by manual and automated extraction, we used as a test case myosin heavy or light chains. For this comparison, we compared the initial automated extraction (*Figshare* file “DIA analysis (2000 most abundant)”, Data Citation 2), which contained 9 myosins, with manual extraction or a second automated search process, each using a spectral library that included not only the DDA developmental time course data, but the entire DDA dataset with whole cochlea, whole utricle, and isolated hair bundles as well as the developmental samples. These data are also available (*Figshare* file “Myosins DIA results”, Data Citation 2). We were able to reliably detect three additional myosin proteins using manual extraction, allowing us to quantify 12 myosins. Using automated extraction with the expanded spectral library, we quantified 19 myosins. In both cases, the increased number of identified proteins highlights the value of using large spectral libraries for analysing DIA data.

**Figure 6:**
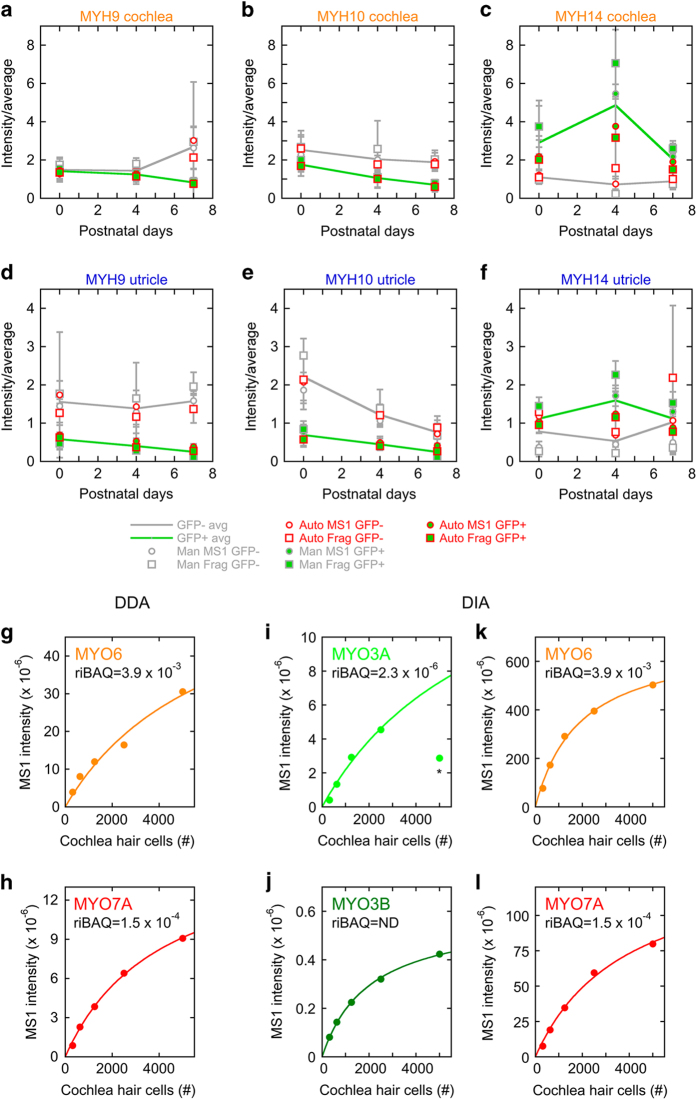
Analysis of myosin heavy and light chains with DIA. **a**–**h**, Conventional myosin heavy chain genes over development using manual and automated data extraction. Fragment-ion and MS1 intensities were extracted from the manual and automated myosins dataset, averages for each peptide and sample condition were calculated across all samples. Only peptides detected in ≥8 samples were used for calculations. The intensity/average ratio was calculated for each peptide and each sample, and these were averaged across the three replicates for each sample condition. The average intensity/average across the three time points was calculated for GFP-positive and GFP-negative samples. Note that the trends were broadly similar for manual and automated extraction, as well as fragment-ion vs. MS1 intensities. **g**-**l**, Detection sensitivity for unconventional myosins in cochlear hair cells. Dilutions of a P0 GFP-positive cochlea sample were prepared (312, 625, 1250, and 2500 cells), and were analysed along with the standard 5000 cell samples. MS1 intensities were measured from DDA experiments (**g,h**) and DIA experiments (**i–l**) for selected myosins, and the sum of intensities was plotted for 2 (MYO3A and MYO3B) or 6 (MYO6 and MYO7A) peptides. Only unique peptides were used in the analysis. The data were fit with y=a1*x/(a2+x); for MYO3A, the final data point (asterisk) was not used in the fit. Each panel also includes the riBAQ (estimated mole fraction) for each protein from the DDA data. MYO3B was not detected in the DDA P0 GFP-positive cochlea samples.

We compared the manual and automated extraction methods using MYH9, MYH10, and MYH14 myosin heavy chains (Fig. 6a–f). Whether using manual or automated extraction, or using fragment-ion or MS1 intensities, the trends for GFP-positive and GFP-negative were very similar across developmental ages. We noted that the coefficients of variation for fragment-ion and MS1 measurements were not statistically different, nor were the coefficients of variation for manual and automated extraction.

### Sensitivity of detection using DDA and DIA

Prior to sample preparation, the 5000 cells in each sample contain ~1 μg of total protein (Materials and Methods). Because one of the expected advantages of DIA quantitation is increased sensitivity for detection of peptides^14^, we examined dilutions of 312-5000 cells to determine lower limits for detection by both DDA (Fig. 6g,h) and DIA (Fig. 6i–l). We examined several unconventional myosins, which are expressed at widely differing levels in hair cells (*Figshare* files “DDA analysis (all proteins)”, “DDA analysis (2 unique)”, “DIA analysis (2000 most abundant)”, and “Myosins DIA results”, Data Citation 2). Remarkably, even the very scarce MYO3A and MYO3B proteins, which were not detected in comparable DDA samples, demonstrated a robust relationship between cell number and MS1 intensity; each protein was easily detected from only 312 cells (Fig. 6j). This relationship saturated similarly for all myosin peptides, suggesting that peptide abundance was not the limiting factor. Instead, this saturation could have been due to saturation of the reverse-phase chromatography column, ion suppression at higher protein concentrations, or saturation of the AGC target.

## Usage Notes

### DDA mass spectrometry data processing

We used the DDA data to generate the spectral library for analysis of the DIA data. In addition, the DDA data are useful for generating hypotheses to be tested with the more quantitative DIA data. Fig. 1b shows how DDA samples were analysed. MaxQuant version 1.5.1.2 software was used for protein identification and quantitation^22^, after editing of the downloaded default MaxQuant contaminants file associated with the MaxQuant download to remove entries of interest (e.g., actin) and to add additional impurities that entered the bundle-purification workflow (e.g., keratins, haemoglobins). Using Andromeda^21^, the DDA data were searched against Ensembl version GRCm38_71 (released April 2013); the Ensembl FASTA file was supplemented with *Xirp2* alternative splice products^38^. Default MaxQuant settings were used, except for the reanalysis that specified each protein must have two or more unique peptides supporting the identification. "Match between runs" was not used, and protein identifications were reported with a false discovery rate (FDR) of 1%.

We used MaxQuant to calculate iBAQ, a measure of protein abundance. The iBAQ value is obtained by dividing protein intensities by the number of theoretically observable tryptic peptides between 6 and 30 amino acids^29^, and is on average highly correlated with protein abundance^29,39^. As described previously^40^, we used a custom Mathematica version 10 program to further process the MaxQuant "proteinGroups.txt" file. This program (1) replaced default protein names and symbols with user-defined entries; (2) deleted all entries marked by MaxQuant as a "Potential contaminant" or "Reverse" (proteins labelled as "Only identified by site" were retained); (3) grouped together proteins that share >20% of their peptides; and (4) prepared an output file. The output file was imported into Excel, where we (5) calculated relative iBAQ (riBAQ)^31,39^, which is the iBAQ for a protein or protein group (calculated by MaxQuant) divided by all non-contaminant, non-reversed iBAQ values for a replicate; (6) determined means and standard deviations for each experimental condition; (7) determined GFP+/GFP- ratios for each developmental age; and (8) calculated relative enrichment at each developmental age.

### DIA mass spectrometry data processing

DIA data are useful for examining relative expression of peptides and proteins between samples. Fig. 1b shows how DIA samples were analysed. DIA data were analysed with Skyline using a MaxQuant spectral library derived from batch #1 of the FACs cell DDA runs (24 total). Signal extraction was performed on centroided MS1 and MS/MS data with a mass accuracy set to 10 ppm. Ions were filtered such that only +2, +3, +4 and +5 precursor ions and +1 and +2 y and b fragment ions (adjusting transition settings to select from fragment ion 3 to the last ion) were extracted. Ion match tolerance was set to 0.05 m/z and the top six fragment ions were extracted from each library spectrum. The iRT calculator was created using CiRT standard peptides to perform retention time predictions, and all results were imported using retention-time filtering to within 5 min of the predicted time. Peptides with up to two missed cleavages and from 7 to 50 amino acids in length were selected from the library for extraction. The 1872 most abundant proteins with two or more unique peptides were selected from the DDA runs as targets for DIA data extraction. Decoys with reversed sequences were created for all peptides and an mProphet scoring model was created using the data as outlined in the advanced peak picking option within Skyline. The scoring model was trained in ten iterations on the data; specific mProphet feature scores and score distributions have been tabulated (*Figshare* file “mProphet features (DIA model)”, Data Citation 2) and can be visualized in Suppl. Figure 4. Quantification was performed by integrating the MS1 and fragment ion peak areas in Skyline for those precursor ions with a detection Q value less than 0.01 (corresponding to an FDR of 1%) and with total MS1 and fragment ion areas greater than zero in at least one replicate.

For the analysis of myosin proteins, automated analysis was done as above. For manual analysis, all peptides for each protein that were contained in the spectral library were imported into the file. The chromatograms for each peptide were manually inspected and the 5–6 peptides with the highest fragment ion intensities were chosen for each protein. Peak boundaries were adjusted for each sample. If no peak was detected for either the precursors or the correct fragment ions within 5 min of the correct retention time, then no peak was selected and areas were marked as zero. Any fragment ions or precursor isotopes that interfered with the main peak were removed from the document. The total MS1 and fragment ion areas for all peptides in the document were then measured and exported into Excel.

### Code availability

Software packages for MaxQuant (http://www.coxdocs.org/doku.php?id=maxquant:start) and Skyline (https://skyline.ms/project/home/software/Skyline/begin.view) are freely available. The custom Mathematica code that takes the MaxQuant output and groups related proteins was released in ProteomeXchange dataset PXD002167.

## Additional information

**How to cite this article**: Krey, J. F. *et al*. Mass spectrometry quantitation of proteins from small pools of developing auditory and vestibular cells. *Sci. Data* 5:180128 doi: 10.1038/sdata.2018.128 (2018).

**Publisher’s note**: Springer Nature remains neutral with regard to jurisdictional claims in published maps and institutional affiliations.

## Supplementary Material



Supplementary information

## Figures and Tables

**Table 1:** Samples for DDA mass spectrometry.

Sample name	Source tissue	GFP status	Develop-mental age	Number of cells	Ear equivalents	Number of replicates (batch #1)	Number of replicates (batch #2)
C GFP- P0	Cochlea	Negative	P0	5000	n/a	2	2
C GFP+ P0	Cochlea	Positive	P0	5000	n/a	2	2
U GFP- P0	Utricle	Negative	P0	5000	n/a	2	0
U GFP+ P0	Utricle	Positive	P0	5000	n/a	2	0
C GFP- P4	Cochlea	Negative	P4	5000	n/a	2	0
C GFP+ P4	Cochlea	Positive	P4	5000	n/a	2	0
U GFP- P4	Utricle	Negative	P4	5000	n/a	2	2
U GFP+ P4	Utricle	Positive	P4	5000	n/a	2	2
C GFP- P7	Cochlea	Negative	P7	5000	n/a	2	0
C GFP+ P7	Cochlea	Positive	P7	5000	n/a	2	0
U GFP- P7	Utricle	Negative	P7	5000	n/a	2	0
U GFP+ P7	Utricle	Positive	P7	5000	n/a	2	0
P0 C57 0pt5 prep1 COCH	C57BL/6 cochlea	n/a	P0	n/a	0.5	1	0
P0 C57 0pt25 prep1 COCH	C57BL/6 cochlea	n/a	P0	n/a	0.25	2	0
P0 C57 0pt25 prep2 COCH	C57BL/6 cochlea	n/a	P0	n/a	0.25	0	2
P0 C57 0pt5 prep2 COCH	C57BL/6 cochlea	n/a	P0	n/a	0.5	0	1
P0 C57 0pt5 UTR	C57BL/6 utricle	n/a	P0	n/a	0.5	7	1
P5 CD1 15ear BUN	CD1 utricle	n/a	P5	n/a	15	1	0
P24 CD1 15ear BUN	CD1 utricle	n/a	P24	n/a	15	1	0
0pt25utr P22CD1 UTR	CD1 utricle	n/a	P22	n/a	0.25	1	0

**Table 2:** Samples for DIA mass spectrometry.

Name	Source tissue	GFP status	Developmental age	Number of cells	Number of replicates
C GFP- P0	Cochlea	Negative	P0	5000	3
C GFP+ P0	Cochlea	Positive (hair cells)	P0	5000	3
U GFP- P0	Utricle	Negative	P0	5000	3
U GFP+ P0	Utricle	Positive (hair cells)	P0	5000	3
C GFP- P4	Cochlea	Negative	P4	5000	3
C GFP+ P4	Cochlea	Positive (hair cells)	P4	5000	3
U GFP- P4	Utricle	Negative	P4	5000	3
U GFP+ P4	Utricle	Positive (hair cells)	P4	5000	3
C GFP- P7	Cochlea	Negative	P7	5000	3
C GFP+ P7	Cochlea	Positive (hair cells)	P7	5000	3
U GFP- P7	Utricle	Negative	P7	5000	3
U GFP+ P7	Utricle	Positive (hair cells)	P7	5000	3
C GFP+ P0 312 cells	Cochlea	Positive (hair cells)	P0	312	1
C GFP+ P0 625 cells	Cochlea	Positive (hair cells)	P0	625	1
C GFP+ P0 1250 cells	Cochlea	Positive (hair cells)	P0	1250	1
C GFP+ P0 2500 cells	Cochlea	Positive (hair cells)	P0	2500	1
