# Supplementary material for: Mass spectrometry quantitation of proteins from small pools of developing auditory and vestibular cells
Source: Sci Data. 2018 Jul 17;5:180128. doi: 10.1038/sdata.2018.128 (PMC6049031; doi:10.1038/sdata.2018.128)

Supplemental Information

Mass spectrometry quantitation of proteins from small pools of developing auditory and vestibular cells

Jocelyn F. Krey^1^, Deborah I. Scheffer2, Dongseok Choi3, Ashok Reddy4,5, Larry L. David4,5, David P. Corey2, and Peter G. Barr-Gillespie^1,4^

1Oregon Hearing Research Center & Vollum Institute, Oregon Health & Science University, Portland, OR

2Department of Neurobiology, Harvard Medical School, Boston, MA

3OHSU-PSU School of Public Health, Oregon Health & Science University, Portland, OR; Graduate School of Dentistry, Kyung Hee University, Seoul, Korea

4Department of Biochemistry and Molecular Biology, Oregon Health & Science University, Portland, OR

5Proteomics Shared Resource, Oregon Health & Science University, Portland, OR

**Corresponding author:** Peter Barr-Gillespie (gillespp@ohsu.edu)

Table of Contents

Supplemental Figure Titles and Legends 1

Supplemental Figure 1 2

Supplemental Figure 2 3

Supplemental Figure 3 4

Supplemental Figure 4 5

Supplemental Figure Titles and Legends

**Suppl. Figure 1.** Pairwise comparison of riBAQ values for all 32 DDA samples. Plots of comparisons of riBAQ values for each protein plotted pairwise for each of the DDA samples on log-log scale. Linear fits to the data are displayed with red lines, while the correlation coefficient is listed in the open boxes.

**Suppl. Figure 2.** Pairwise comparison of riBAQ values for 24 batch #1 DDA samples. Plots are the same as for Suppl. Fig. 2, except only the batch #1 samples are shown.

**Suppl. Figure 3.** Pairwise comparison of riBAQ values for 24 batch #1 DDA samples, ≥2 unique peptides. Plots are the same as for Suppl. Fig. 2, except only the batch #1 samples are shown and the data were analysed requiring at least two unique peptides for identification.

**Suppl. Figure 4.** mProphet model description. ***A***, Composite score assigned to each decoy peak (orange) and each target peak (blue) by the model. Most of the target peptides are to the right of the Gaussian fit that describes the decoy distribution. ***B***, Q values of target peptides. ***C***, P values of decoy and target peptides. The uniform distribution of decoy values between 0 and 1 indicates that the decoys are fit well with a Gaussian.


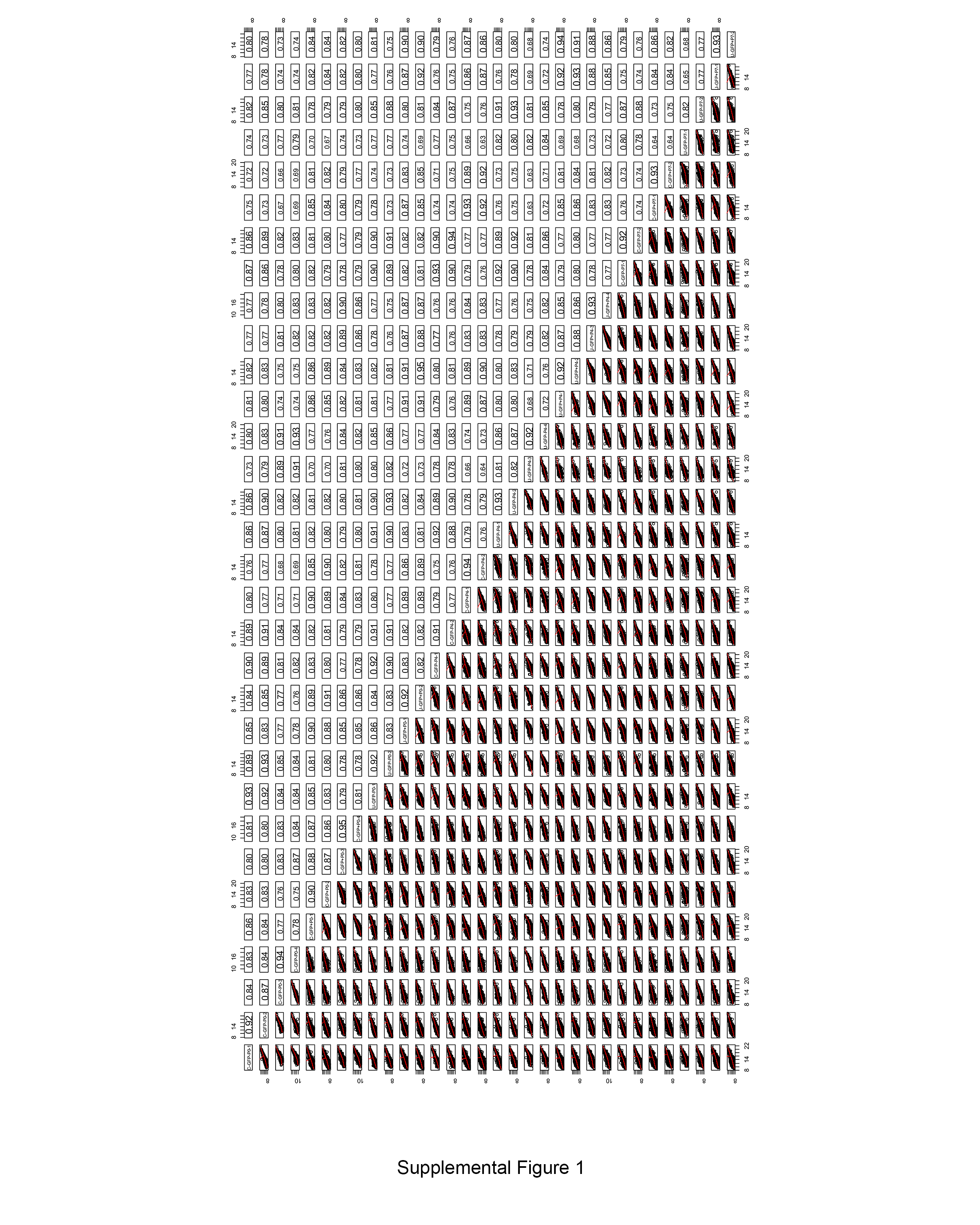


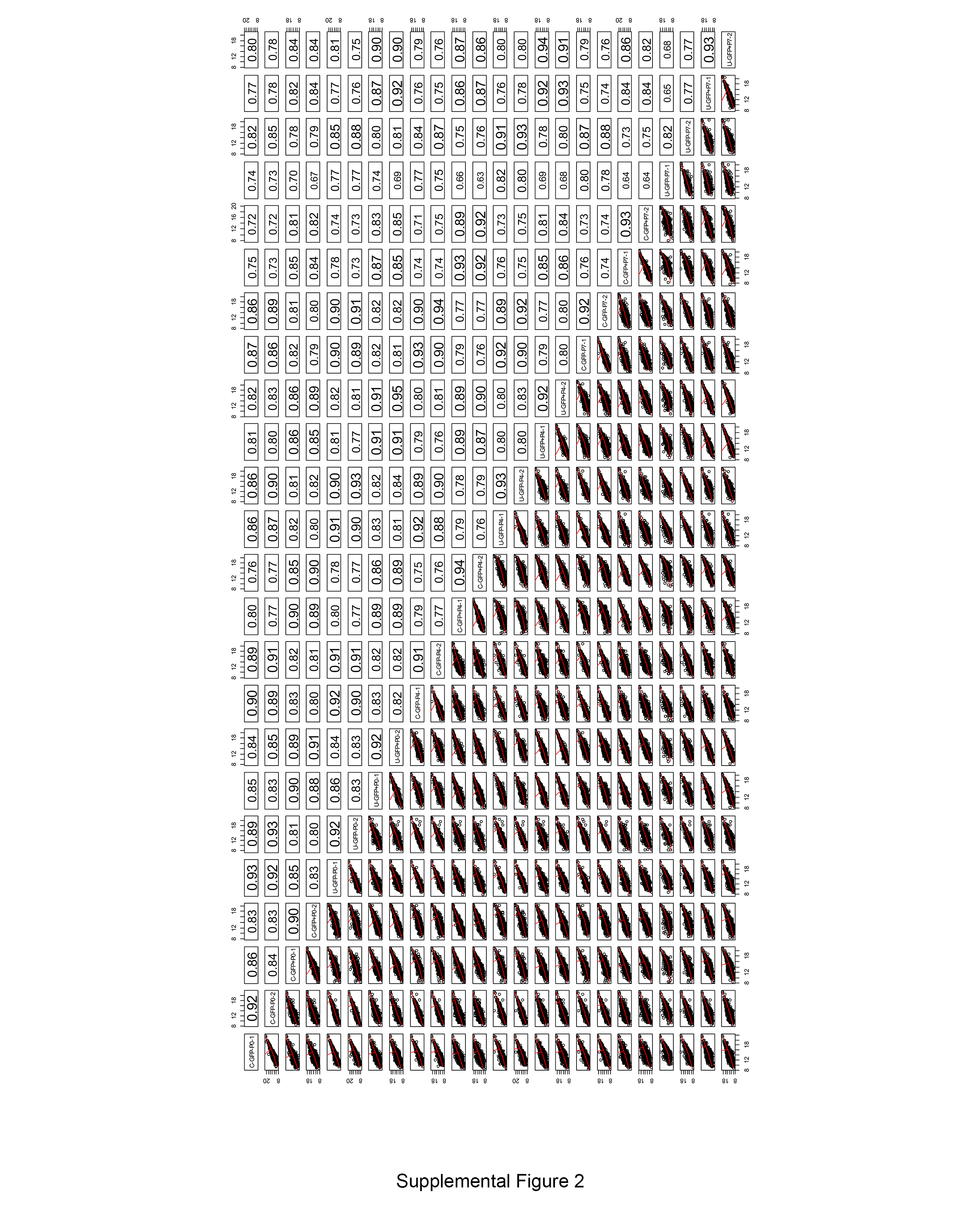


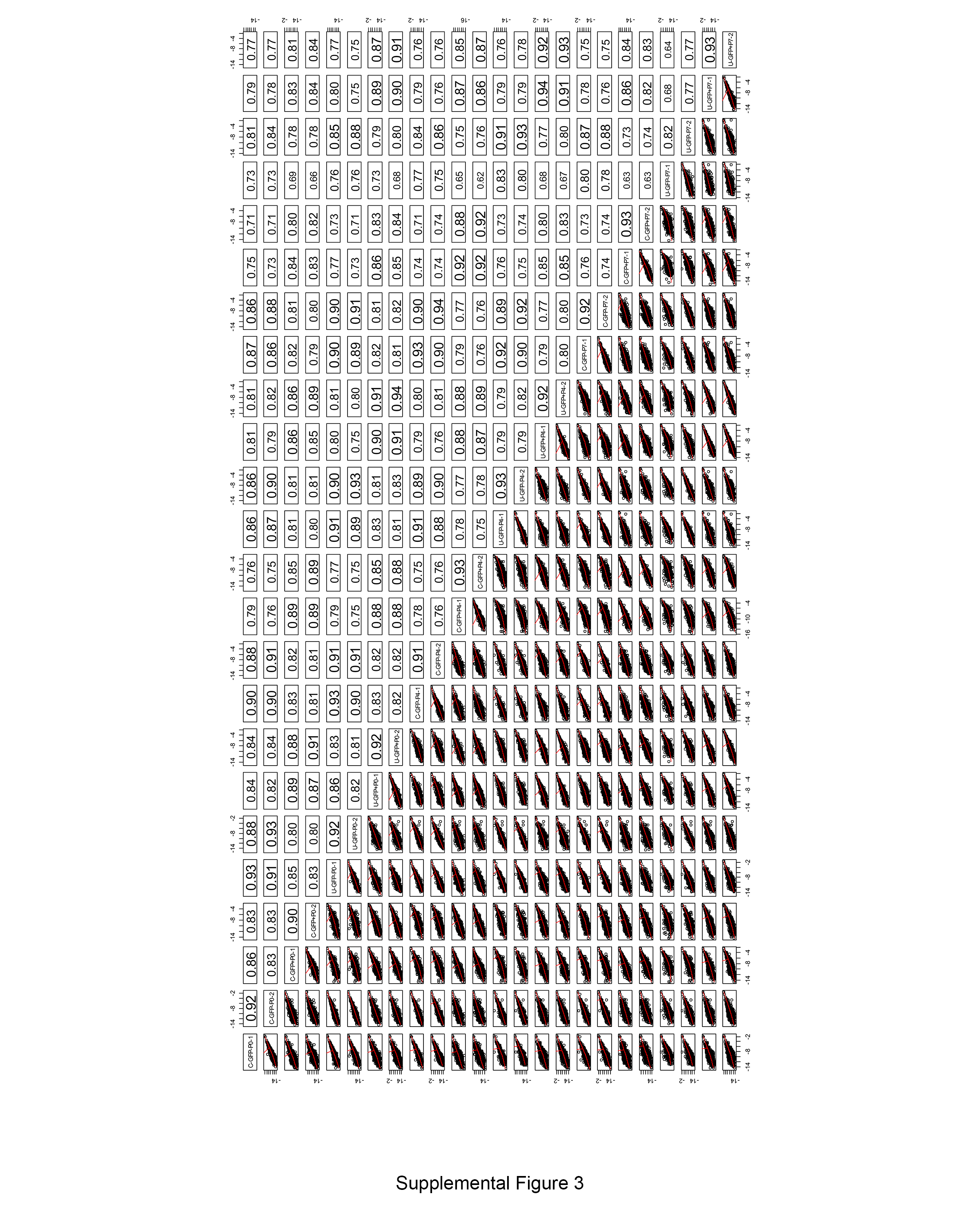


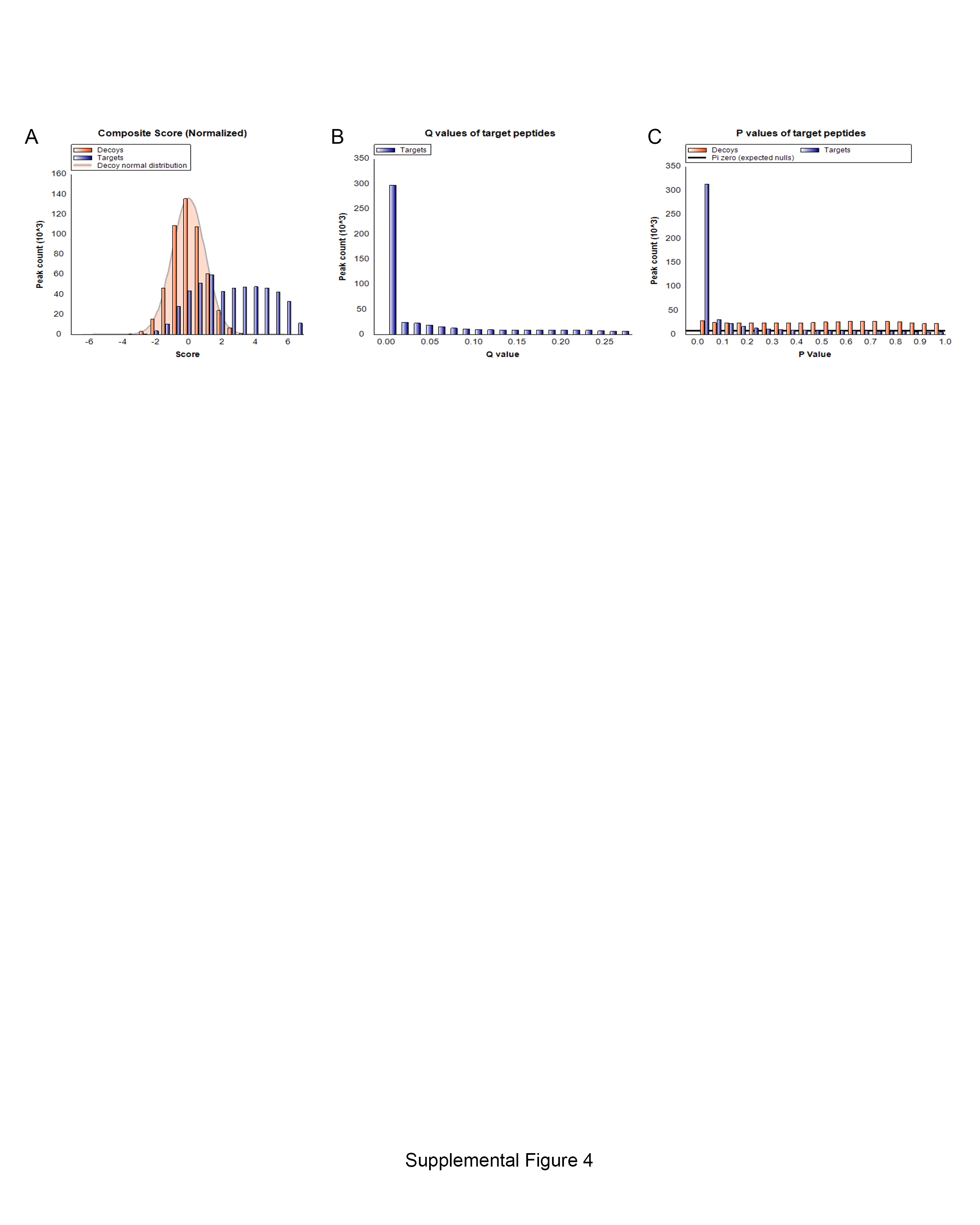

Supplement: Supplementary information [file sdata2018128-s2.docx]
